# Reasons Why Some Japanese Pregnant Women Choose Trial of Labor After Cesarean

**DOI:** 10.14740/jocmr2214w

**Published:** 2015-06-09

**Authors:** Shunji Suzuki, Mariko Ikeda

**Affiliations:** a Department of Obstetrics and Gynecology, Japanese Red Cross Katsushika Maternity Hospital, Tokyo, Japan

**Keywords:** Trial of labor after cesarean, Elective repeat cesarean delivery, Counseling, Japan

## Abstract

**Background:**

We examined whether or not the Japanese pregnant women with a history of a cesarean section have the knowledge about the benefits and harms of trial of labor after cesarean (TOLAC) and elective repeat cesarean delivery (ERCD).

**Methods:**

We reviewed the obstetric records of 121 Japanese women with a prior cesarean section who visited our hospital for reservation of their second delivery between January and December 2013.

**Results:**

Forty-five (37%) of them wanted to perform TOLAC at the first interview. Of these, 14 women (31%) with a history of an urgent cesarean chose TOLAC because of the insufficient anesthetic effect during cesarean, while 11 women (24%) with a history of an elective cesarean did not have the knowledge of the risks of TOLAC and urgent cesarean. Nineteen of those (76%) selected ERCD following the counseling.

**Conclusions:**

Some Japanese pregnant women with TOLAC hope seemed to have insufficient knowledge about the benefits and harms of TOLAC and ERCD. Therefore, the improvement of the process of counseling and decision making may be needed for pregnant women with a history of a cesarean section in Japan.

## Introduction

Labor after previous cesarean delivery has been reported to have a risk of uterine rupture associated with serious perinatal complications of less than 1% [1]. However, the hospital length of stay, incidence of postpartum transfusion and incidence of postpartum fever have been observed to be significantly lower in cases of trial of labor after cesarean (TOLAC) than in cases of elective repeat cesarean delivery (ERCD) [1]. Therefore, women with a prior cesarean should be thoroughly counseled after the first cesarean section and/or during prenatal care about the long-term effects of cesarean such as placenta previa, placenta accrete and abdominal adhesions and the benefits and harms of both a TOLAC and an ERCD, and they should be offered the opportunity to make an informed decision about mode of birth [2-4].

In Japan, a cautious medical policy has been taken against TOLAC as compared to the United States [5]. The rate of vaginal birth after cesarean (VBAC) seemed to be low in Japan. For example, in the 256 facilities registered with the Japan Society of Obstetrics and Gynecology (total number of delivery: about 150,000), there were 653 cases of VBAC in 2012 [6]. In addition, in our institute, which is one of major perinatal centers in Tokyo, Japan, the rate of VBAC was decreased from 33% in 2002 to 10% in 2012 [7].

Based on these backgrounds, we examined whether or not the Japanese pregnant women with a history of a cesarean section have the knowledge about the benefits and harms of TOLAC and ERCD.

## Methods

We reviewed the obstetric records of all Japanese women with a prior cesarean section who visited our hospital for reservation of their second delivery between January and December 2013. An interview was conducted to ask them whether or not they hope to perform TOLAC at their first visits. If the women hope to perform TOLAC, an additional interview concerning the reason for TOLAC hope and the counseling about the benefits and harms of both a TOLAC and an ERCD were conducted. Demographic information about their previous cesarean was extracted from patient charts.

Data are presented as number (%). For statistical analysis, the Χ^2^ test was used. Differences with P < 0.05 were considered significant.

## Results

In 2013, there were 121 Japanese pregnant women with a prior cesarean section who visited our hospital for reservation of their second delivery. Eighty-one of them (67%) had a previous urgent cesarean section, while 40 (33%) had a previous elective cesarean section.

Forty-five (37%) of them wanted to perform TOLAC at the first interview. Of these, 14 women (31%) with a history of an urgent cesarean chose TOLAC because of the insufficient anesthetic effect during cesarean and/or the presence of serious abdominal pain during the 2 - 3 days after cesarean. Eleven of them (79%) selected ERCD following the counseling concerning that a reliable anesthesia and pain relief can be performed during and after scheduled cesarean as compared to emergent cesarean.

On the other hand, 11 women (24%) with a history of an elective cesarean did not have the knowledge of the risks of TOLAC and urgent cesarean. In addition, three of them did not know anything about uterine rupture or the long-term effects of multiple cesareans. Following the counseling, eight of those (73%) selected ERCD.

## Discussion

The counseling and knowledge about the risks and benefits of TOLAC and ERCD are very important and they have been observed to be positively associated with the decision for TOLAC [2]. From the current results, the Japanese women seemed to have insufficient knowledge about the risk of emergent anesthesia and/or TOLAC. In this study, we did not examine the reason in cases selected ERCD; however, it seems to be evident that some Japanese pregnant women with a history of a cesarean did not seem to receive the sufficient description of the risks and benefits of TOLAC and ERCD. In our institute, the incidence of intrauterine fetal demise and low umbilical artery pH was significantly decreased, and a negative correlation was found between the cesarean delivery rate and the incidence of low umbilical artery pH for each year [7]; however, the improvement of the process of counseling and decision making may be needed for pregnant women with a history of a cesarean section in Japan.

In conclusions, some Japanese pregnant women with TOLAC hope seemed to have insufficient knowledge about the benefits and harms of TOLAC and ERCD.
